# High Temperature-Induced Oxidative Stress Affects Systemic Zinc Homeostasis in Broilers by Regulating Zinc Transporters and Metallothionein in the Liver and Jejunum

**DOI:** 10.1155/2022/1427335

**Published:** 2022-03-28

**Authors:** Chuanpi Xiao, Linglian Kong, Xue Pan, Qidong Zhu, Zhigang Song, Nadia Everaert

**Affiliations:** ^1^Department of Animal Science, Shandong Agricultural University, Taian, Shandong 271018, China; ^2^Precision Livestock and Nutrition Unit, Gembloux Agro-Bio Tech, University of Liège, Gembloux 5030, Belgium

## Abstract

To investigate the change in zinc homeostasis of broilers under heat stress, 512 broiler chickens were raised to the age of 28 days. The broilers were then assigned to heat stress and normal temperature (36.0°C vs. 26.0°C) groups for 7 days. The results showed that oxidative stress induced by high temperature had a negative effect on the growth performance of broilers. Heat stress altered zinc homeostasis and led to a redistribution of zinc in broilers, which was reflected in increased zinc concentrations in the jejunum, liver, and tibia. Upregulation of the expression of the zinc exporter *ZnT1* and importers *ZIP8* and *ZIP14* in the jejunum indicated that more zinc was absorbed and transported from the jejunum into the blood, while the liver increased its capacity to hold zinc through upregulation of metallothionein (*MT*) expression, which was achieved by reducing *ZnT1* expression and upregulating the expression of the importer *ZIP3*. The pathway was mediated by zinc transporters, but the capacity of *MT* to chelate and release zinc ions also played a crucial role. The mechanism of alterations in zinc homeostasis under heat stress was revealed by the changes in zinc transporters and *MT* levels in the intestine and liver. Heat stress also altered cecal microbial diversity and reduced the relative abundances of *Bilophila* and *Dialister*. In conclusion, broilers altered systemic zinc homeostasis through the regulation of zinc transporters and *MT* in the liver and jejunum to resist oxidative stress induced by high temperature.

## 1. Introduction

With global warming and the aggravation of the greenhouse effect, heat stress has become a challenge that cannot be ignored in animal husbandry. In poultry production, broilers have shown great growth potential with the improvement of modern breeding technology [1]. However, the increased growth rate will accelerate metabolism and likely lead to heat stress; the lack of sweat glands in broilers is not conducive to heat dissipation. Heat stress could cause physiological system disorders in broilers, including immune system damage, respiratory alkalosis, and hormone secretion disorders [2]. These phenomena could lead to oxidative stress by affecting mitochondrial function and changing reactive oxygen species (ROS) levels, thereby causing oxidative damage to proteins and lipids and changes in the levels of oxidative stress markers, such as malondialdehyde (MDA), glutathione peroxidase (GPX), and superoxide dismutase (SOD) [3, 4]. When heat stress occurs, the heat shock protein (HSP) family begins to act as a stress indicator and cell protector [5]. A large number of studies have shown that the *HSP70* protein is related to the immune function of birds, such as controlling the transcription of various genes in response to immune stimulation by regulating the activity of nuclear factor kappa light chain enhancer of B cells (*NF-κB*) [6].

Zinc, an essential trace element, participates in a variety of enzymatic reactions and affects various biological processes, such as digestion, absorption, and metabolism of nutrients in animals [7]. Therefore, the metabolism and homeostasis of zinc is a complex physiological process. An increasing amount of evidence indicates that many protein families, such as metallothionein (*MT*) and zinc-regulated transporters (Zrts), iron-regulated transporter- (Irt-) like proteins, and zinc transporters, play major roles in zinc metabolism and homeostasis [8]. *MT* is a family of low-molecular metal-binding proteins that are rich in cysteine and usually bind zinc in cells to serve as a zinc reservoir [9]. *MT* is not only a zinc reservoir but also a potential diagnostic biological marker of intracellular zinc content. *MT* also participates in a variety of physiological processes, including oxidative stress and immune function [10]. Zrt- and Irt-like proteins (*ZIPs*) belong to the SLC39A family and transport zinc into the cytoplasm from extracellular or cellular organelles; zinc transporters (*ZnTs*) belong to the SLC30A family and transport zinc from the cytoplasm to the outside of the cell, functioning in zinc mobilization across biological membranes. Thus far, 10 ZnT transporters and 14 ZIP transporters have been discovered [11]. In an inflammatory or oxidative stress state, the level of hepatic *MT* can rise rapidly, while the expression of the zinc finger protein *A20* is altered; moreover, *NF-κB* is negatively regulated to inhibit the production of inflammatory cytokines [12].

Adding zinc to feed has positive effects on animal growth and immunity. According to the National Research Council (NRC, 1994), the requirement of zinc for broilers is 40.0 ppm; approximately 100 ppm zinc from different sources is typically added to feed to optimize animal performance [13, 14]. The underlying mechanisms could be attributed to altered intestinal histomorphology and reduced inflammation and oxidative stress [15, 16] resulting in improved growth of broilers. Increasing lines of evidence indicate that zinc is redistributed in animals under conditions of oxidative stress and immune challenge [17, 18]. However, changes in zinc homeostasis in broilers under heat stress conditions and the regulatory mechanism have not been reported.

In this study, we investigated the changes in systemic zinc homeostasis and the regulatory mechanism in broilers under oxidative stress and inflammation caused by heat stress. The role of zinc transporters and metallothioneins in the regulation of zinc metabolism under heat stress conditions was innovatively revealed.

## 2. Materials and Methods

All experimental procedures were approved by the Ethics Committee of Shandong Agricultural University and performed in accordance with the Guidelines for Experimental Animals of the Ministry of Science and Technology (Beijing, People's Republic of China). All feeding and euthanasia procedures were performed with full consideration of animal welfare.

### 2.1. Experimental Design and Management

A total of 512 28-day-old male broiler chicks (Arbor Acre) with similar weights were randomly divided into two treatment groups, each of which included 16 replicates (cages) and 16 birds per cage. The two treatments were as follows: half of the birds were assigned to temperature treatment at 36.0 ± 1°C (heat stress group, HT) for 7 days; birds in the other treatment group were raised at a normal temperature of 26.0 ± 1°C (CON). All birds were given free access to pellet feed and water during the rearing period. The basal diet composition and nutrition levels of the basal diet are listed in Table 1. During the first 3 days, the average relative humidity was maintained at approximately 70.0% and was maintained between 55.0% and 65.0% thereafter. In the first week of life, the birds were given 23 h of light and 1 h of darkness; this schedule was gradually changed to 20 h of light and 4 h of darkness by the end of the 35-day test. All broilers were vaccinated by means of drinking water. Newcastle disease (ND) and infectious bronchitis (IB) vaccines were administered on day 6 and infectious bursal disease (IBD) vaccine on day 12. The use of antibiotics was strictly prohibited to ensure the effectiveness of the test.

### 2.2. Growth Performance

At 35 days of age, feed consumption was recorded to calculate the average daily feed intake (ADFI) for each replicate. The birds were weighed to calculate the average daily gain (ADG). The feed conversion ratio (FCR) was defined as ADFI : ADG. Mortality data were recorded and included in the FCR calculation.

### 2.3. Sample Collection

One bird was randomly selected from each cage at 35 days of age after the measurement of growth performance. Blood samples were collected intravenously with a sterile syringe from the wing, placed in a glass tube without anticoagulant, and centrifuged at 3000 rpm at 4°C for 10 min after being left to stand for 30 min. Serum was obtained and stored at -20°C for biochemical analysis. The birds were euthanized by cervical dislocation after obtaining blood samples. Approximately 2 cm segments were excised from the midjejunum (from the entry point of the bile duct to Meckel's diverticulum), flushed repeatedly with a cold saline solution, and immediately immersed in a 4% paraformaldehyde solution for histological examination. Tissue samples of approximately 1 g to 2 g were collected from the jejunum and liver, rapidly frozen in liquid nitrogen, and stored at -80°C for further analysis. Tibia from both sides were dissected carefully and stored at -20°C until analysis. All birds selected were fasted for eight hours before the procedure.

### 2.4. Analysis of Oxidative Stress and Cytokines in Serum

The total SOD (T-SOD) activity and MDA, aspartate aminotransferase (AST), and alanine aminotransferase (ALT) levels were measured in the serum by using diagnostic kits purchased from Nanjing Jiancheng Biotechnology Institute (Nanjing, China). All determination procedures were performed strictly according to the manufacturer's instructions. The intra-assay coefficient of variation (CV) was less than 5%, and the interassay CV was less than 8%.

Endotoxin, interleukin 1*β* (IL-1*β*), interleukin 4 (IL-4), interleukin 6 (IL-6), interleukin 10 (IL-10), and tumor necrosis factor-*α* (TNF-*α*) in serum were detected using ELISA kits (MLBIO Co., Shanghai, China) according to the manufacturer's instructions. The inter- and intra-assay CVs were less than 10%.

### 2.5. Zinc and Metallothionein Concentrations

The serum concentration of zinc was measured by an inductively coupled plasma optical emission spectroscopy instrument (ICAP 7000, Thermo, USA). Each serum sample (75 *μ*L) was placed in a centrifuge tube with 600 *μ*L of HNO_3_ (5%) and 75 *μ*L of hydrogen peroxide (30%). The centrifuge tubes were placed in a water bath at 60°C for 2 h, and 750 *μ*L of HNO_3_ (5%) was added. The mixed solution was centrifuged for 15 min (8000 r/min), and the supernatant was stored at 4°C until measurement. The concentrations of zinc in the tibia, liver, jejunum, and cecal contents were evaluated via flame atomic absorption spectrometry (SpectrAA50/55, Warman Corporation, Palo Alto, CA, USA). The tibiae were boiled in deionized water for 10 min, soaked in ether for 96 h, degreased, and dried at 105°C to constant weight. The liver and jejunum tissues were freeze dried for 24 h and weighed. The samples from the tibia, liver, and jejunum were ashed in a muffle oven (550-600°C for 24 h). Ash content was measured and expressed as dry degreased weight. The ash of the sample was dissolved with 0.6 mol/L hydrochloric acid and filtered. The solution was stored at 4°C before determination.

The concentrations of *MT* in the liver and jejunum were measured using ELISA kits (MLBIO Co., Shanghai, China) according to the manufacturer's instructions. The inter- and intra-assay CVs were less than 10%.

### 2.6. Total RNA Extraction and Real-Time PCR

Total RNA in the jejunum was extracted with TRIzol reagent (Invitrogen, San Diego, USA). The concentration and purity of each RNA sample were detected using a NanoDrop spectrophotometer (ND-2000, Thermo Scientific, Wilmington, USA). RNA integrity was detected by 1% agarose gel electrophoresis. Reverse transcription of 1 *μ*g of total RNA was performed using a PrimeScript® RT reagent kit (RR047A, TaKaRa, Japan). RT-PCR analysis was performed to determine gene expression by using TB Green Premix Ex Taq (RR820A, Takara, Japan) in an ABI 7500 Real-Time PCR System (Thermo Scientific, Wilmington, USA). The reaction program included the following: predenaturation at 95°C for 10 s, followed by 40 cycles of denaturation at 95°C for 5 s and annealing and extension at 60°C for 40 s. Each reaction was repeated in triplicate wells, and the primer sequences are shown in Table 2. The amplification efficiencies of the primers were calculated using a standard curve. The specificities of the amplified products were verified by the melting curve. The geometric mean of the expression of *β-actin* and glyceraldehyde-3-phosphate dehydrogenase (*GAPDH*) was used to normalize the expression of the target genes. Relative gene expression levels of each target gene were analyzed using the 2^−ΔΔct^ method.

### 2.7. Western Blot Analysis

Frozen liver and jejunum samples were crushed into powder in liquid nitrogen, and protein extraction was performed using a total protein extraction kit (CW Biotech, Beijing, China) as specified by the manufacturer's instructions. After the protein concentration of the sample was determined using the BCA protein analysis kit (Beyotime Institute of Biotechnology, Beijing, China), a 40 *μ*g protein sample was loaded into a 10% sodium dodecyl sulfate-polyacrylamide gel for electrophoresis (New Cell & Molecular Biotech Co., Suzhou, China). The protein was separated in electrophoresis buffer at a constant voltage of 150 mV and then transferred to a polyvinylidene difluoride (PVDF) membrane (Invitrogen, Carlsbad, CA, USA) with transfer buffer (Beyotime Institute of Biotechnology, Beijing, China) at a constant current of 400 mA. After membrane transfer, the PVDF membrane was immersed in blocking buffer for 0.5 h and then incubated overnight with primary antibodies at 4°C. Primary antibodies against *ZnT1* (1 : 500) and *GAPDH* (1 : 2000) were purchased from Bioss Technology Inc. After three washes with TBST buffer, a secondary horseradish peroxidase-conjugated antibody (Beyotime Institute of Biotechnology, Beijing, China) was incubated at room temperature for 1 h. Finally, the protein blot strips were visualized in a gel imager using ECL kits (Beyotime Institute of Biotechnology, Beijing, China), and signals were quantified using the measurement software in the imager. *GAPDH* protein was used as an internal control for all the immunoblotting bands to obtain the corresponding expression of the target proteins.

### 2.8. 16S rRNA Gene Amplicon Sequencing

Samples of cecal contents were collected on ice after slaughter and immediately stored in a -80°C freezer for subsequent analysis. Microbial DNA extraction from cecum contents was performed according to the instructions of the E.Z.N.A.® soil kit (Omega Bio-Tek, Norcross, GA, USA). The DNA concentration and purity were measured using a NanoDrop 2000 spectrophotometer, and the quality of DNA extraction was determined using 1% agarose gel electrophoresis. PCR amplification of the V3-V4 variable region of the bacterial 16S rRNA gene was performed using the 338F and 806R primers. The PCR conditions consisted of an initial denaturing program for 3 min at 95°C, 27 cycles (95°C for 30 s, 55°C annealing for 30 s, and 72°C for 30 s), and a final extension step at 72°C for 10 min (PCR instrument: GeneAmp9700 produced by ABI). PCRs were performed in a 20 *μ*L mixture: 4 *μ*L of 5X FastPfu buffer, 2 *μ*L of 2.5 mM dNTPs, 0.8 *μ*L of primer (5 *μ*M), 0.4 *μ*L of FastPfu polymerase, and 10 ng of DNA template. PCR products were recovered using a 2% agarose gel, purified using an AxyPrep DNA Gel Extraction Kit (Axygen Biosciences, Union City, CA, USA) and quantified using QuantiFluor™-ST (Promega, USA).

Purified amplicons were pooled in equimolar amounts and paired-end sequenced (2 × 300) on an Illumina MiSeq platform (Illumina, San Diego, USA) according to standard protocols. Quantitative Insights Into Microbial Ecology 2 (QIIME2) software was used for quality screening of the raw sequences and quality filtering and pruning, denoising, merging, and chimera removal of the demultiplexed sequence of each sample were carried out to obtain the amplicon sequence variation feature table. According to the 338F/806R primers, the database obtained in the previous step was pruned to the V3-V4 region to obtain the species classification table. After all contaminating mitochondrial and chloroplast sequences were removed, appropriate methods, including ANCOM, ANOVA, Kruskal-Wallis test, and LEfSe, were used to identify bacteria with differential abundances between samples and groups. QIIME2 core diversity was used to calculate the horizontal alpha diversity index of feature sequences, including observed operational taxonomic units (OTUs), Chao1 abundance estimator, Shannon diversity index, and Faith's phylogenetic diversity index. The beta diversity index included Bray-Curtis, unweighted UniFrac, and weighted UniFrac indices, which were used to evaluate the structural changes in microbial communities between samples and are presented in principal coordinate analysis (PCoA) and nonmetric dimensional scaling (NMDS) diagrams. Partial least squares discriminant analysis in R software was used as a monitoring model to reveal the relationship between the microbial community and sample category. The R package “Vegan” redundancy analysis method was used to reveal potential associations between microbial communities and related environmental factors. Spearman rank correlation coefficients were calculated using cooccurrence analysis to show associations between species based on the relative abundances of major microbial species in the samples. The parameters used in the analysis were set as the defaults.

### 2.9. Statistical Analysis

Data are presented as the mean ± SD. All data were analyzed through normal distribution determination. Growth performance was analyzed on a replicate (per cage) basis while other indicators were analyzed on an individual basis. Differences in the treatments were analyzed with a *t*-test in SPSS 22.0 (SPSS. Inc., Chicago, USA). Significance was set as *P* < 0.05.

## 3. Results

### 3.1. Growth Performance

**Figure 1:**
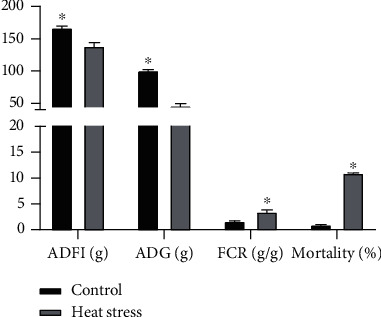
Effects of heat stress on growth performance. ADG: average daily gain; ADFI: average daily feed intake; FCR: feed conversion ratio.

Heat stress had a significant effect on growth performance. As shown in Figure 1, heat stress significantly reduced the ADFI and ADG (*P* < 0.05) and increased the FCR and mortality of 35-day-old broilers (*P* < 0.05).

### 3.2. Analysis of Oxidative Stress and Cytokines in Serum

The heat stress group had higher (*P* < 0.05) T-SOD and endotoxin levels in serum (Table 3). No significant difference was found in serum MDA levels (*P* > 0.05) between the heat stress and normal temperature groups. The levels of serum IL-4, IL-6, and IL-10 increased (*P* < 0.05) under heat stress. No significant effect was observed in serum IL-1*β* and TNF-*α* levels (*P* > 0.05) between the heat stress and normal temperature groups.

### 3.3. Zinc Concentration in Tissues and Cecal Contents

**Figure 2:**
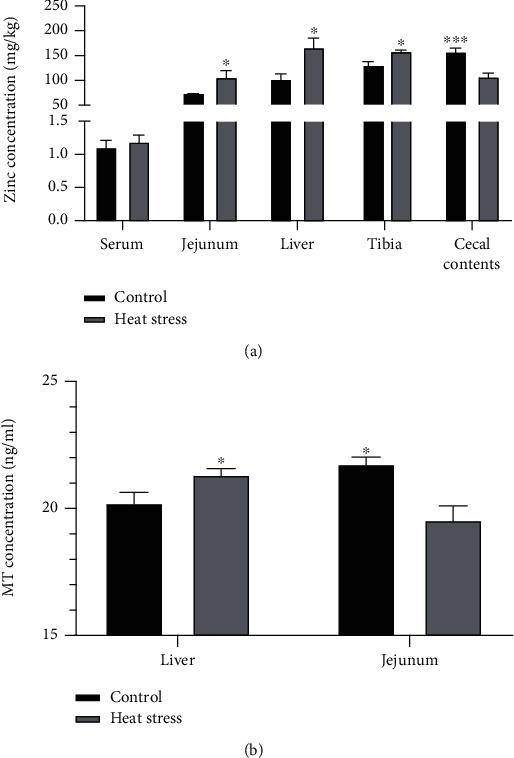
Zinc and metallothionein concentrations in different tissues of broilers were affected by heat stress. (a) Zinc concentrations in the serum, jejunum, liver, tibia, and cecal contents of broilers; (b) *MT* concentrations in the liver and jejunum of broilers. *MT*: metallothionein.

As shown in Figure 2(a), the concentration of zinc increased (*P* < 0.05) in the jejunum, liver, and tibia in response to heat stress, while the opposite result was observed in the cecal contents. However, heat stress had no significant effects (*P* > 0.05) on the serum concentration of zinc. The results indicated that zinc was redistributed under heat stress.

### 3.4. *MT* Concentration in Tissues

Figure 2(b) summarizes the *MT* concentration in the liver and jejunum. Heat stress increased (*P* < 0.05) the *MT* concentration in the liver. Moreover, the *MT* concentration in the jejunum was decreased under heat stress conditions (*P* < 0.05).

### 3.5. mRNA Expression of Jejunal and Hepatic Genes

**Figure 3:**
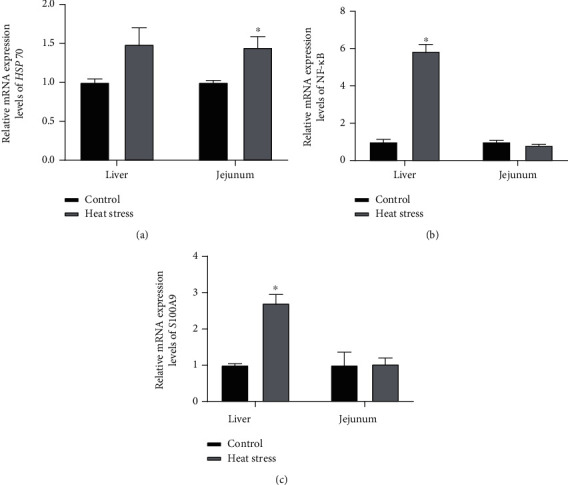
Influence of heat stress on the gene expression of *HSP70* (a), *NF-κB* (b), and *S100A9* (c) in the liver and jejunum. *HSP70*: heat shock protein 70; *NF-κB*: nuclear factor kappa light chain enhancer of B cells.

Figure 3(a) shows the mRNA expression of *HSP70* in the jejunum and liver after heat stress. We also determined the mRNA expression levels of the immune and inflammatory genes calprotectin *NF-κB* and *S100A9* (Figures 3(b) and 3(c)). In general, the expression of the *HSP7*0 gene in the jejunum was upregulated after heat stress (*P* < 0.05). Moreover, the expression levels of *S100A9* and *NF-κB* in the liver were upregulated (*P* < 0.05).

**Figure 4:**
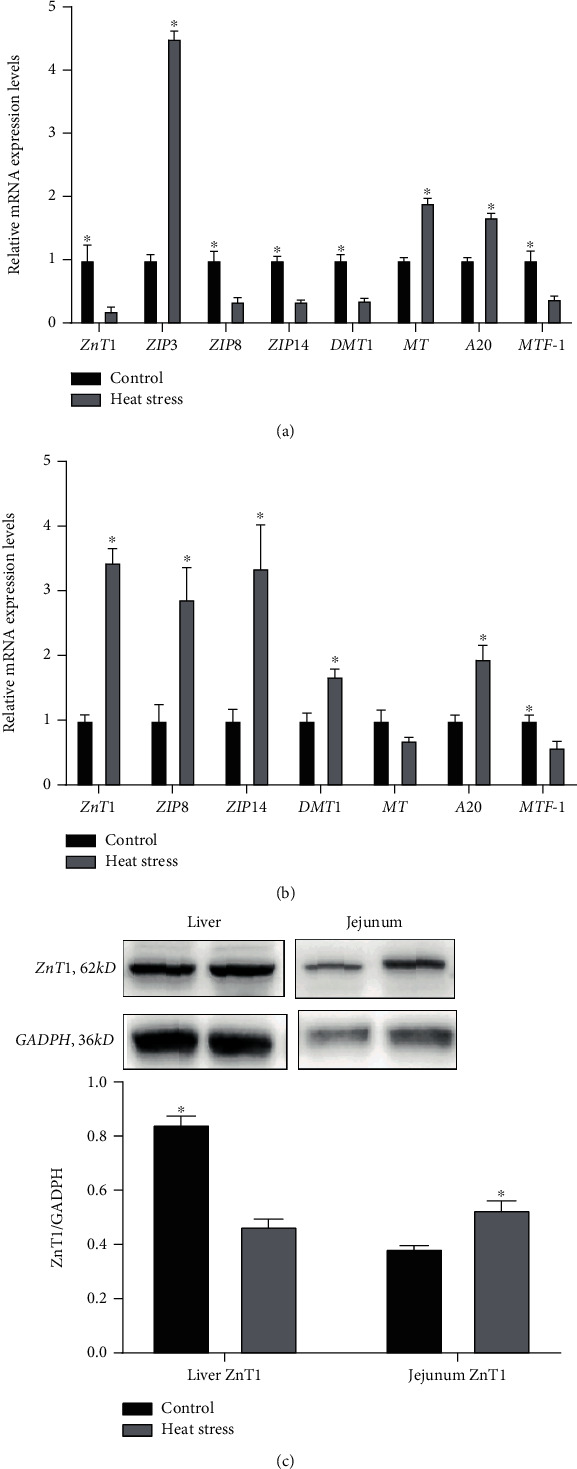
Influence of heat stress on gene and protein expression in the liver and jejunum. (a) Zinc transporter- and zinc transporter regulation-related genes in the liver; (b) zinc transporter- and zinc transporter regulation-related genes in the jejunum; (c) protein expression of *ZnT1* in the liver and jejunum. *MT*: metallothionein; ZIP: zinc-regulated transporter, iron-regulated transporter-like protein; ZnT: zinc transporter; *DMT1*: divalent metal transporter 1; *MTF-1*: metal transcription factor-1.

Figure 4 shows that heat stress altered zinc metabolism in the liver and jejunum, which plays a crucial role in regulating systemic zinc homeostasis. The expression of the zinc transporters *ZnT1*, *ZIP8*, and *ZIP14* was downregulated (*P* < 0.05), and that of the divalent metal transporter 1 (*DMT1*) showed the same trend (*P* < 0.05) in the liver. The mRNA expression of the zinc importer *ZIP3* was significantly upregulated (*P* < 0.05) in the livers of heat-stressed broilers (Figure 4(a)). Moreover, heat stress increased the gene expression levels (*P* < 0.05) of zinc transporters (*ZnT1, ZIP8*, and *ZIP14*) and *DMT1* in the jejunum (Figure 4(b)). Heat stress resulted in higher gene expression levels of *MT* and the zinc finger protein *A20* (*P* < 0.05) in the liver, and *A20* expression showed the same trend in the jejunum. The expression of metal-binding transcription factor-1 (*MTF-1*) was downregulated (*P* < 0.05) in both organs.

### 3.6. Protein Expression of Jejunal and Hepatic Genes

As shown in Figure 4(c), heat stress elevated the protein expression of *ZnT1* in the liver but decreased the protein expression of *ZnT1* in the jejunum (*P* < 0.05). This result confirmed the same trend as the gene expression results.

### 3.7. Composition and Community Diversity of the Cecal Microbiota

**Figure 5:**
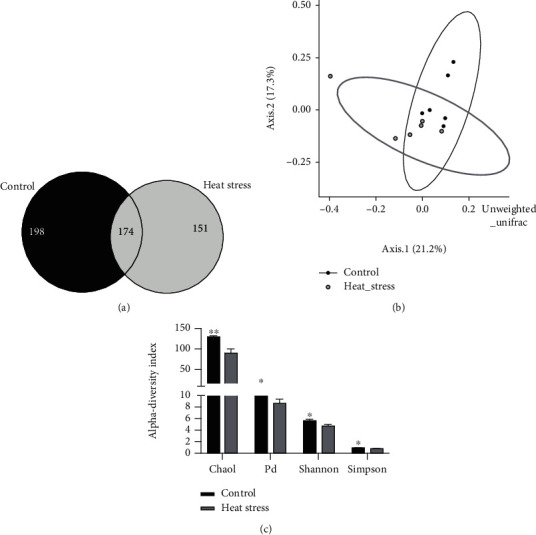
Heat stress altered the community diversity of the cecal microbiota. (a) Venn diagram based on the OTU level. (b) PCoA plots of *β* diversity based on OTUs. (c) *α*-Diversity based on the Chao1, PD, Shannon, and Simpson indices.

In 35-day-old broilers, 174 OTUs were identical in the cecal contents of both the heat stress group and normal temperature group, while 198 and 151 OTUs were unique to the normal temperature group and heat stress group, respectively (Figure 5(a)). *β*-Diversity analysis using unweighted UniFrac distance did not show specific clustering between the two treatments (Figure 5(b)). As shown in Figure 5(c), heat stress significantly decreased the Chao1, PD, Shannon, and Simpson indices of *α* diversity (*P* < 0.05).

**Figure 6:**
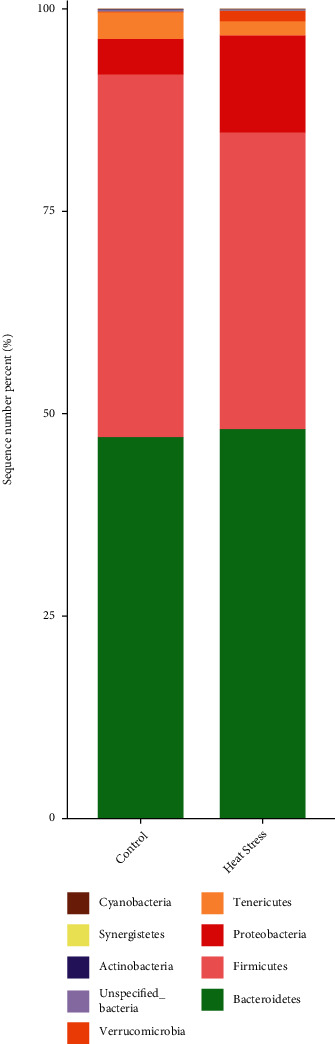
Average relative abundances of predominant bacteria at the phylum level in cecal contents.

**Figure 7:**
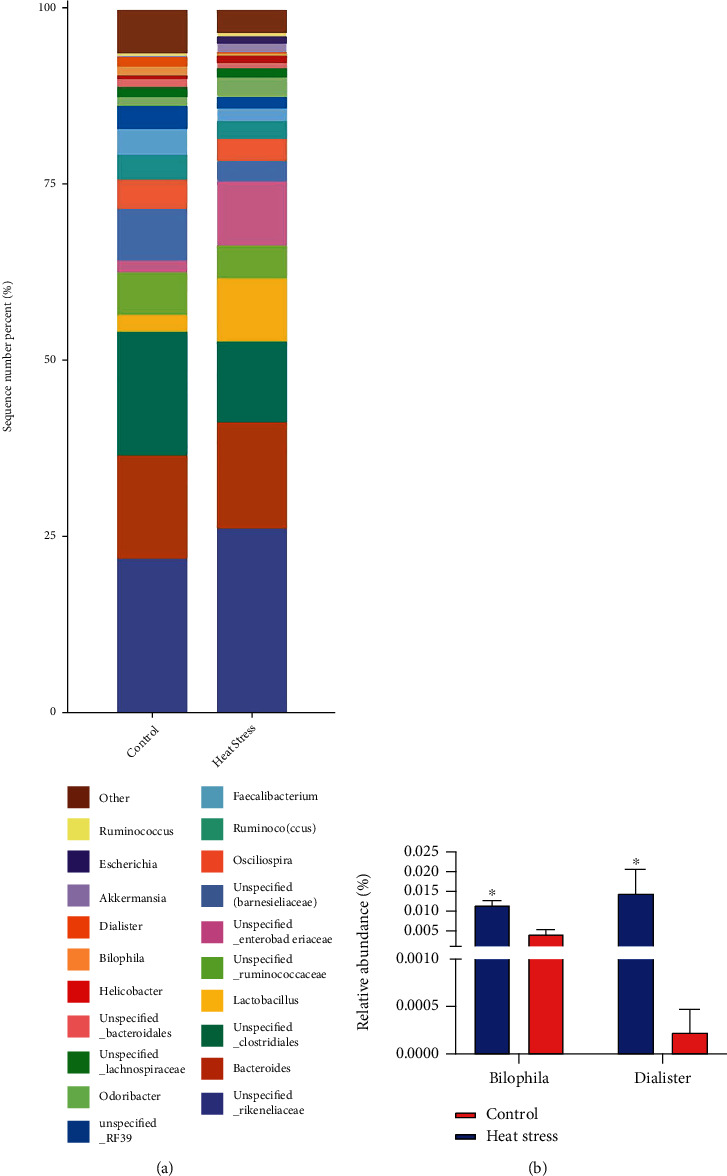
Alteration of the cecal microbiota at the genus level. (a) Average relative abundances of predominant bacteria (top 20) at the genus level in cecal contents. (b) Relative abundances of *Bilophila* and *Dialister*.

Phylum level-based analyses showed that over 95% of the cecal microbiota in both treatment groups was dominated by three major phyla: *Bacteroidetes*, *Firmicutes*, and *Proteobacteria* (Figure 6). There was no significant effect of heat stress on the relative abundance of the cecum microbial community at the phylum level. Taxonomic analysis of the relative abundances of the 20 predominant genera in each group was used to confirm specific changes in the microbial community (Figure 7(a)). The results showed that heat stress reduced the relative abundances of *Bilophila* and *Dialister* at the genus level (Figure 7(b)).

## 4. Discussion

Heat stress causes a variety of physiological changes, such as oxidative stress and immune function suppression, leading to increased mortality and decreased feed efficiency, body weight, and feed intake [19, 20]. In the present study, heat stress decreased broiler performance, as manifested by the decrease in ADFI and ADG. High temperatures increased mortality in broilers from 1% to 11%, while feed conversion rates more than doubled. The effect of heat stress has been reported to be responsible for the decrease in feed intake as well as layer weight, feed utilization, and egg production and quality [21]. Birds need to breathe to release heat at high ambient temperatures. A previous study indicated that eating is restricted during heat stress because birds cannot simultaneously breathe and eat. Therefore, birds spend more time panting than eating when exposed to high temperatures [22]. Another reason is that hyperthermic birds aim to reduce metabolic heat by eating less to decrease heat generation [23].

High temperatures can cause oxidative stress. The levels of MDA and T-SOD can effectively assess the antioxidant balance of animals [24]. The activity of antioxidant enzymes in the liver and serum significantly increase with increasing environmental temperature [25]. In the present study, heat stress increased the T-SOD content in broiler serum as a protective response to oxidative stress [26]. Exposure to acute heat stress (35.0°C, 3 h) resulted in increased liver lipid peroxidation in broilers. However, heat stress did not affect the serum MDA level of broilers, which may be related to the duration of exposure to heat stress. Under heat stress conditions, the blood flow distribution changes from the visceral capillaries to the peripheral capillaries, resulting in a rapid drop in body temperature [27]. However, reduced visceral blood flow may lead to hypoxia in gastrointestinal tissues [28], oxidative stress damage, and increased permeability to pathogens and related endotoxins [29]. Heat stress increases the serum endotoxin (ET) content, suggesting increased intestinal permeability.

HSP is a protein chaperone in cells that senses oxidative damage and plays a role in cell protection under various stresses [30]. Consistent with the present study, previous work has reported that the expression of *HSP70* increased in the presence of oxidative stress [31]. The *NF-κB* pathway is a ubiquitous participator in the expression of proinflammatory cytokines, such as IL-1*β*, IL-6, IL-8, and TNF-*α*, after an inflammatory challenge with lipopolysaccharide (LPS) [32]. Heat stress can activate the expression of *NF-κB* in the liver and jejunum of birds, leading to the secretion of a series of proinflammatory cytokines [33, 34]. Calprotectin is also a biomarker of inflammation and belongs to the S100 protein family, whose expression is elevated in the gut and liver following inflammatory infection [35]. In the present study, the expression levels of *NF-κB* and *S100A9* were upregulated in the liver, similar to those of *HSP70* in the jejunum. Hence, oxidative stress caused by heat stress can change the expression of immune regulatory genes and inflammatory marker genes and eventually lead to an increase in the serum levels of anti-inflammatory cytokines (IL-4, IL-6, and IL-10).

As a classical type 2 nutrient, zinc should be ingested frequently, and its homeostasis must be regulated accurately [36]. After heat stress, zinc in the liver was redistributed, and *MT* gene expression in the liver was upregulated, indicating that the liver increased zinc storage in response to oxidative stress, as demonstrated by the increase in zinc content in the liver, tibia, and jejunum. In the carrier-mediated process of zinc absorption [37], zinc can be absorbed into intestinal epithelial cells through the enterocyte apical membrane by *DMT1* and ZIP family transporters [38] and then stored for binding with metallothionein. The zinc exporter *ZnT1* is located at the basolateral membrane and contributes to the transport of zinc from the intestine into the portal vein circulation. The liver plays an important role in zinc homeostasis due to its rich content and fast exchange in the metabolism of zinc [39]. Zinc uptake by the liver is necessary for the plasma through the regulation of transporter proteins. The importer *ZIP3* is involved in the transport of zinc from serum to the liver, where zinc is stored in cells after binding to metallothionein and transported out of the liver by the exporter *ZnT1* [40].

Our results suggested that zinc was redistributed in the jejunum and liver after heat stress. The expression levels of zinc importers (*DMT1*, *ZIP8*, and *ZIP14*) and the exporter *ZnT1* increased in the jejunum, indicating that more zinc was absorbed from the intestinal tract as evidenced by a decrease in the concentration of zinc in the intestinal contents. More interestingly, the expression of *ZIP3* increased and that of *ZnT1* decreased, which meant that the liver needed more zinc reserves, as reflected by the higher contents of liver *MT* and zinc. Heat stress altered zinc homeostasis in chickens, as reflected in the increased zinc contents in tissues and bone. The adjustment of zinc transporter levels in different organs played an incredible role in increasing the ability of zinc to be absorbed in the intestine, while the liver, as a major organ of metabolism, had an increased ability to store zinc. Oxidative stress and immune challenge process is accompanied by a decrease in serum zinc concentration and an increase in liver zinc concentration providing antioxidant defense system [41]. Similar to the results after seven days of Salmonella infection in broilers [42], we did not find a decrease trend in serum zinc concentrations after heat stress, which should be attributed to the adaptation of broilers to temperature and adequate zinc content in the feed.

Accumulating evidence shows that oxidative stress can alter the expression of zinc transporters [43]. *ZIP14* directly or indirectly promotes intestinal zinc absorption and regulates zinc metabolism in response to inflammatory stimuli in the liver [44]. Oxidative stress induced by chronic alcohol exposure can downregulate the expression of the zinc transporter *ZIP14* in mouse hepatocytes [45]. In aged cardiomyocytes, the *ZIP8* level has been found to decrease with increasing ROS levels [46]. *ZIP8* and *ZIP14* are configured differently from the other members of the ZIP family because a histidine is replaced by glutamic acid [47], which may be the key to their immunity and antioxidant roles. As the only zinc-sensitive transcription factor, *MTF-1* is involved in zinc concentration sensing and *MT* regulation [48]. *MT* not only regulates the concentration of free zinc in cells but also affects the production of ROS in cells to resist stress by regulating the expression of *NF-κB* [49]. Zinc concentration affects the expression of the zinc finger protein *A20*, which in turn changes the ability of *NF-κB* to assemble with DNA and reduces the inflammatory response caused by oxidative stress [50]. Heat stress decreased the expression of *MTF-1* and increased the expression of *A20* in the liver and jejunum, indicating that the increase in the tissue zinc concentration downregulated the expression of the sensing gene *MTF-1*, while the zinc finger protein *A20* activated the anti-inflammatory effect of *NF-κB*. These modifications support the physiological significance of greater accumulation of zinc in tissues for the promotion of zinc-mediated antioxidant actions.

The cecal microbiota resists inflammatory bowel disease and metabolic disorders and helps maintain normal barrier function and antioxidant capacity [51, 52]. Our results confirm that heat stress alters the microbial composition of the broiler cecum and that lower levels of intestinal microbes may be associated with the negative effects of heat stress on intestinal health and microbial community stability. We did not find significant correlations between heat stress and the cecum microbiota at the phylum level, similar to previous findings in yellow-feathered broilers [53]. At the genus level, heat stress treatment reduced the relative abundances of *Bilophila* and *Dialister*. *Bilophila* is involved in the anaerobic metabolic pathway that converts the substrate taurine, which is abundant in the gut microbiota, into the toxic metabolite hydrogen sulfide. A reduction in the relative abundance of *Bilophila* predicts a weakening of intestinal immune stimulation and the inflammatory response after heat stress treatment [54]. In the gut microbiota of pigs fed higher levels of lysine-chelated zinc diets, *Dialister* had a high relative abundance, promoting the decarboxylation of succinate to propionate [55]. Propionic acid is a short-chain fatty acid (SCFA), and zinc can result in the enrichment of beneficial SCFAs by increasing the abundance of corresponding bacteria in the gut, thereby influencing the composition of the microbiota to promote zinc absorption by the host [56]. In our study, heat stress enhanced intestinal absorption of zinc via transporter proteins, leading to a low-zinc environment in the gut and resulting in a decrease in the relative abundance of *Dialister*. Therefore, the reduction in cecum microbial abundance in broiler chickens due to heat stress is associated with an attenuated inflammatory response and a low-zinc environment in the intestine.

## 5. Conclusions

Heat stress could cause oxidative stress and immune challenge in broilers, which can be reflected in the decrease in production performance. Our results confirmed the remodeling of zinc homeostasis in broilers under heat stress and identified the role of zinc transporters and metallothionein in the liver and jejunum during this process. The effects of heat stress on the gut microbiota seem to be involved in the redistribution of zinc, which will be further explored in future studies.

## Figures and Tables

**Table 1:** Composition and nutritional levels of the basal diet.

Item	1-21 days	22-35 days
Ingredient (%)		
Corn	49.98	54.9
Soybean meal (46%)	35.75	30.5
Corn protein flour (60%)	3.80	3.10
Salt	0.280	0.280
Limestone	1.75	1.62
Dicalcium phosphate	1.55	1.40
Soybean oil	5.10	6.50
Vitamin premix	0.050	0.050
Mineral premix	0.200	0.200
Choline chloride (50%)	0.100	0.100
Methionine (99%)	0.350	0.350
Lysine (70%)	0.800	0.750
Threonine (98.5%)	0.290	0.250
Phytase (20000 U)	0.020	0.020
Total	100	100
Nutritional level		
Metabolizable energy	3100 (kcal/kg)	3200 (kcal/kg)
Crude protein	23.5	21.0
Lysine	1.39	1.20
Methionine+cystine	1.02	0.920
Calcium	1.00	0.900
Total phosphorus	0.990	0.900
Available phosphorus	0.500	0.450

Provided per kilogram of compound diet: vitamin A: 12000 IU; vitamin D3: 5000 IU; vitamin E: 80 mg; vitamin K: 3.2 mg; vitamin B1: 3.2 mg; vitamin B2: 8.6 mg; nicotinic acid: 65 mg; pantothenic acid: 20 mg; vitamin B6: 4.3 mg; biotin: 0.22 mg; folic acid: 2.2 mg; vitamin B12: 0.017 mg; I: 1.50 mg; Fe: 80 mg; Mn: 120 mg; Se: 0.3 mg; Cu: 16 mg; and Zn: 110 mg. The nutrition level was calculated.

**Table 2:** Nucleotide sequences of real-time PCR primers.

Gene	Accession number	Primer sequence, 5′→3′	Product size (bp)
*ZnT1*	NM_001389457.1	F: CTTCGCTTAGCATTTCTT	75
		R: TCTCCGATTTAGTCCTTCT	
*DMT1*	NM_001128102.2	F: AGCCGTTCACCACTTATTTCG	129
		R: GGTCCAAATAGGCGATGCTC	
*ZIP3*	NM_144564.4	F: GGGCACTTTCTTGTTCATCACC	105
		R: GCAGCATAACCCAGCACCAG	
*ZIP8*	XM_040671236.1	F: TGTAAATGTCTCGGTGGG	159
		R: CAAGATGGCTATGGAGGT	
*ZIP14*	XM_040689606.1	F: GTTCTGCCCCGCTGTCCT	96
		R: GGTCTGCCCTCCTCCGTCT	
*MT*	NM_205275.1	F:GCAACAACTGTGCCAAGGGC	138
		R: TTTCGTGGTCCCTGTCACCC	
*MTF-1*	XM_015297695.3	F: CCTGGTTCAACTCCTATGC	278
		R: TCAAACGGCTTCTCCTTA	
*NF-λB*	NM_205129	F:GTGTGAAGAAACGGGAACTG	203
		R: GGCACGGTTGTCATAGATGG	
*A20*	XM_003640919.2	F:GACATCGTGCTAACAGCTTGGA	141
		R: AGAAAAGAGGTATCAGGCACAAC	
*S100A9*	NM_001305151.1	F: TTGAGAAGCAGCTTGCCAACTAC	187
		R: TGCTGTTGCTGGTGGTCCTC	
*HSP70*	NM_001006685.1	F: TCTCATCAAGCGTAACACCAC	104
		R: TCTCACCTTCATACACCTGGAC	
*β-Actin*	NM_205518.1	F: ATGTGGATCAGCAAGCAGGAGTA	127
		R: TTTATGCGCATTTATGGGTTTTGT	
*GADPH*	NM_204305	F: ACATGGCATCCAAGGAGTGAG	266
		R: GGGGAGACAGAAGGGAACAGA	

*MT*: metallothionein; *ZIP*: zinc-regulated transporter, iron-regulated transporter-like protein; *ZnT*: zinc transporter; *DMT1*: divalent metal transporter 1; *MTF-1*: metal transcription factor-1; *NF-κB*: nuclear factor kappa-B; *HSP70*: heat shock protein 70; *GAPDH*: glyceraldehyde-3-phosphate dehydrogenase.

**Table 3:** Effects of heat stress on serum parameters and cytokine levels.

Temperature	Control	Heat stress	*P* value
MDA (nmol/mL)	3.11 ± 0.127	3.09 ± 0.274	0.9491
T-SOD (U/mL)	100 ± 7.48^b^	120 ± 5.27^a^	0.0472
Endotoxin (EU/mL)	0.317 ± 0.0338^b^	0.469 ± 0.0484^a^	0.0153
IL-1*β* (ng/L)	28.0 ± 3.50	29.4 ± 2.68	0.7442
IL-4 (ng/L)	129 ± 7.08^b^	219 ± 25.1^a^	0.0012
IL-6 (ng/L)	63.8 ± 8.64^b^	118 ± 11.0^a^	0.0011
IL-10 (ng/L)	100 ± 9.84^b^	182 ± 18.1^a^	0.0001
TNF-*α* (ng/L)	171 ± 12.1	158 ± 11.3	0.4471

MDA: malondialdehyde; T-SOD: total superoxide dismutase; IL-4: interleukin 4; IL-6: interleukin 6; IL-10: interleukin 10; IL-1*β*: interleukin 1*β*; TNF-*α*: tumor necrosis factor-*α*. Values are expressed as the mean ± SD (*n* = 8). Different superscripts (a, b) in the same line indicate significant differences (*P* < 0.05).

## Data Availability

The 16S rRNA sequence data used to support the findings of this study have been deposited in the GenBank repository (PRJNA770158).

## Abbreviations

ROS: Reactive oxygen species
MDA: Malondialdehyde
GPX: Glutathione peroxidase
T-SOD: Total superoxide dismutase
*HSP*: Heat shock protein
*NF-κB*: Nuclear factor kappa light chain enhancer of B cells
MT: Metallothionein
ZIP: Zinc-regulated transporter:
ZnT: Zinc transporter, iron-regulated transporter-like protein
ADFI: Average daily feed intake
ADG: Average daily gain
FCR: Feed conversion ratio
AST: Aspartate aminotransferase
ALT: Alanine aminotransferase
CV: Coefficient of variation
IL-1*β*: Interleukin 1*β*
IL-4: Interleukin 4
IL-6: Interleukin 6
IL-10: Interleukin 10
TNF-*α*: Tumor necrosis factor-*α*
*GADPH*: Glyceraldehyde-3-phosphate dehydrogenase
*DMT1*: Divalent metal transporter 1
*MTF-1*: Metal transcription factor-1
PVDF: Polyvinylidene difluoride
OTUs: Operational taxonomic units.
